# The Effects of *in* *Situ*-Formed Silver Nanoparticles on the Electrical Properties of Epoxy Resin Filled with Silver Nanowires

**DOI:** 10.3390/polym8040157

**Published:** 2016-04-21

**Authors:** Gwang-Seok Song, Dai Soo Lee, Ilho Kang

**Affiliations:** 1 Division of Semiconductor and Chemical Engineering, Chonbuk National University, Baekjedaero 567, Deokjin-gu, Jeonju, Chonbuk 54896, Korea; michaelis@naver.com; 2 Research Center, NEPES AMC, 99 Seokam-ro, Iksan, Chonbuk 54587, Korea

**Keywords:** *in situ*-formed silver nanoparticles, silver nanowire, epoxy resin, nanocomposite

## Abstract

A novel method for preparing epoxy/silver nanocomposites was developed via the *in situ* formation of silver nanoparticles (AgNPs) within the epoxy resin matrix while using silver nanowires (AgNWs) as a conductive filler. The silver–imidazole complex was synthesized from silver acetate (AgAc) and 1-(2-cyanoethyl)-2-ethyl-4-methylimidazole (imidazole). AgNPs were generated *in situ* during the curing of the epoxy resin through the thermal decomposition of the AgAc–imidazole complex, which was capable of reducing Ag^+^ to Ag by itself. The released imidazole acted as a catalyst to cure the epoxy. Additionally, after the curing process, the *in situ*-generated AgNPs were stabilized by the formed epoxy network. Therefore, by using the thermal decomposition method, uniformly dispersed AgNPs of approximately 100 nm were formed *in situ* in the epoxy matrix filled with AgNWs. It was observed that the nanocomposites containing *in situ*-formed AgNPs exhibited isotropic electrical properties in the epoxy resins in the presence of AgNWs.

## 1. Introduction

Nanocomposite materials containing metal nanoparticles, graphenes, or organoclays dispersed into a polymer matrix exhibit significant absorbing properties because of their novel physical and chemical properties [1,2,3]. Specifically, epoxy/metal nanocomposites were used in the field of microelectronic packaging for applications in embedded capacitors and lead-free interconnecting materials [4,5,6,7]. One-dimensional nanostructured particles such as nanowires, nanotubes, nanorods, or nanofibers are expected to play an important role in fabricating nanoscale devices and nanocomposites. Metal nanowires such as silver nanowires (AgNWs) were employed as conductive fillers [8,9,10,11,12,13,14,15,16]. AgNWs have very low electrical resistivity and high thermal conductivity. Consequently, AgNWs have been primarily used as conductive fillers in conductive adhesive materials. An appropriate distribution of AgNWs within the epoxy network is very important for these applications. However, AgNWs are thermodynamically unstable and tend to form agglomerates because of their surface activity. Agglomeration occurs, and the anisotropic performance of AgNWs by the aspect ratio is a significant problem when NWs are used to fabricate composites with high performance. Polymeric stabilizing agents, such as poly(vinylpyrrolidone), poly(ethyleneglycol), and other long-chain acids or amines, are usually introduced in the chemical syntheses of AgNWs to prevent agglomerate formation [17,18,19,20,21,22,23,24,25]. However, these stabilizing agents are seldom removed from the AgNW surfaces because of the strong interaction between them. These residues of organic molecules on NW surfaces are harmful, as they reduce electrical resistivity. Therefore, their application in electronic packaging is limited [26].

A uniform dispersion of AgNWs in nanocomposites is required as clumps of wires inside the polymer matrix lead to undesirable electrical, dielectric, and thermal properties. However, it is difficult to achieve uniformly dispersed ultrafine NWs in a polymer matrix by incorporating pre-added AgNWs into a polymer. This is because of the easy agglomeration of NWs and high viscosity of polymer systems with AgNWs. Conversely, the *in situ* formation of silver nanoparticles (AgNPs) in a polymer matrix could facilitate a more uniform dispersion of AgNWs in polymers. Further, *in situ* reduction can result in much smaller particle sizes than the commercially available micron or nano size silver particles. This, in turn, can help achieve electrical conductivity for the encapsulants and adhesives.

In this study, a novel method for preparing epoxy/silver nanocomposites was investigated. The method involved forming *in situ* AgNPs via the decomposition of a silver–imidazole complex with AgNWs and the curing of an epoxy resin. At a suitable curing temperature, the AgNP was formed *in situ* in the nanocomposites of the epoxy resin and in the AgNW by the decomposition of the silver–imidazole complex, which was capable of reducing Ag^+^ to Ag by itself. Imidazole was immediately released from the silver–imidazole complex and could act as an epoxy resin catalyst. Specifically, the anisotropic properties of the nanocomposites due to the high aspect ratio of AgNWs disappeared because of the *in situ*-formed AgNP.

## 2. Materials and Methods

### 2.1. Materials

Silver nitrate (AgNO_3_), chloroplatinic acid (H_2_PtCl_6_·6H_2_O), poly(vinylpyrrolidone) (PVP), anhydrous ethylene glycol (EG), and sodium chloride (NaCl) were purchased from Sigma Aldrich Chemical (Yongin, Kyeonggi-do, Korea) for the syntheses of AgNWs. A cycloaliphatic-type epoxy resin was purchased from Daicel Chemical Industry (Minato-ku, Tokyo, Japan). The anhydride-type curing agent, hexahydro-4-methylphthalic anhydride, and silver acetate (AgAc) were purchased from Sigma Aldrich Chemical. An imidazole-type catalyst, 1-(2-cyanoethyl)-2-ethyl-4-methylimidazole (imidazole), was purchased from TCI Chemical (Chuo-ku, Tokyo, Japan). The synthesized AgNW was purified using the washing method. Isopropyl alcohol (IPA), purchased from SK Chemical (Seongnam-si, Kyeonggi-do, Korea), was chosen as the solvent because of its low boiling point and capacity as a good stabilizer for AgNWs.

### 2.2. Synthesis of Silver Nanowires (AgNWs) Using a Microwave Process

The microwave polyol process used in this study was the same as that in the literature [27]. The solution containing AgNO_3_, H_2_PtCl_6_·6H_2_O, PVP (average molecular weight: 55,000 g/mol), and NaCl in EG was irradiated using a microwave oven (LG electronics, Yeongdeungpo-gu, Seoul, Korea) in continuous wave mode at 700 W. The solution was rapidly heated within 2 min, and an AgNW was formed. The AgNW was washed with acetone and dispersed in IPA.

### 2.3. Preparation of Silver–Imidazole Complex

Silver acetate (AgAc, 3 mmol) was added to imidazole (6 mmol). A paste mixer was used to dissolve the mixture until all the solid AgAc had dissolved. The liquid product had a yellow color and was viscous at 25 °C.

### 2.4. Preparation of Epoxy/Silver Nanocomposites with AgNWs

**Scheme 1:**
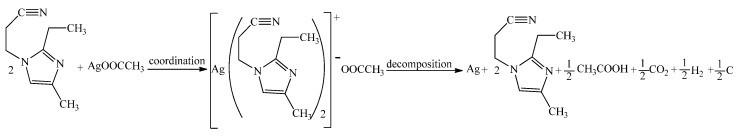
Scheme for the silver nanoparticles (AgNPs) formed by *in situ* thermal decomposition of silver–imidazole complex.

The 10 wt % of silver–imidazole complex was dispersed in the epoxy resin. The molar ratio of AgAc to imidazole was maintained at 1:2. A purified AgNW in IPA was added to the above mixture and was mixed until a light-yellow solution was formed. The solvent was evaporated under reduced pressure at 40 °C. Finally, the mix was cured at 110 °C for 30 min, 150 °C for 2.5 h, and 180 °C for 1 h to obtain epoxy/silver nanocomposites. The thermal decomposition of silver–imidazole complex realized the formation of AgNPs. The silver–imidazole complex was capable of reducing Ag^+^ to Ag under the stepwise curing condition of the epoxy. Scheme 1 shows the synthesis process of AgNPs via the decomposition of the silver–imidazole complex [28]. This was confirmed using thermogravimetric analysis (TGA), field electron scanning electron microscopy (FE-SEM), and energy-dispersive X-ray spectroscopy (EDX) analysis. This study investigated various epoxy/silver nanocomposites with different AgNW loading levels.

The released imidazole in Scheme 1 acted as a catalyst for the curing of the epoxy resin. It was confirmed in DSC that the scanning of the epoxy resin containing anhydride-type curing agent and the silver–imidazole complex accompanied an exotherm due to the cure reaction, while the epoxy resin with only the anhydride-type curing agent without the silver–imidazole complex did not, as shown in Figure S1 in Supplementary Materials.

### 2.5. Characterization

The morphology of AgNWs was recorded using a transmission electron microscope (JEM-2010, JEOL, Akishima, Tokyo, Japan) and scanning electron microscope (JSM-5900, JEOL). The standard copper grid for the TEM image was dipped in the dispersion of a 0.1% AgNW in IPA for transmission electron microscopy (TEM) imaging. The fracture surfaces of the nanocomposite samples were observed under a field emission scanning electron microscope (S-4800, HITACHI, Minato-ku, Tokyo, Japan) after cryogenic fracture. The electrical properties of the nanocomposites were studied by measuring the surface resistivity of the composite sheets with a resistivity meter (ST-4, SIMCO JAPAN, Inc., Chuo-ku, Kobe, Japan) at room temperature. The TGA samples (Q50, TA Instruments, New Castle, DE, USA) were heated at a rate of 10 °C/min up to 800 °C under a nitrogen atmosphere to measure the amount of *in situ*-formed AgNPs.

## 3. Results and Discussion

**Figure 1:**
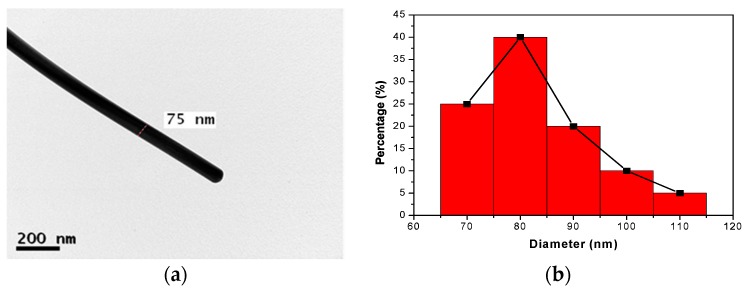
Synthesized silver nanowires (AgNWs): (**a**) transmission electron microscopy image; (**b**) diameter distribution of the purified AgNW.

**Figure 2:**
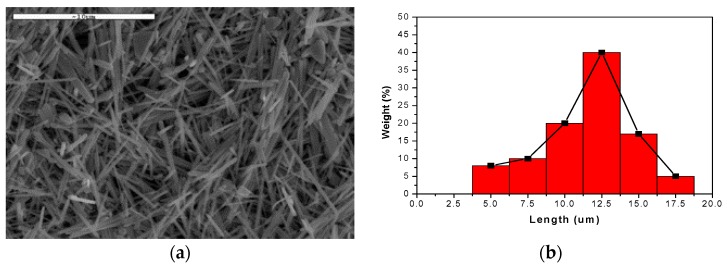
Purified silver nanowires (AgNWs): (**a**) scanning electron microscopy image; (**b**) length distribution of a purified AgNW.

**Figure 3:**
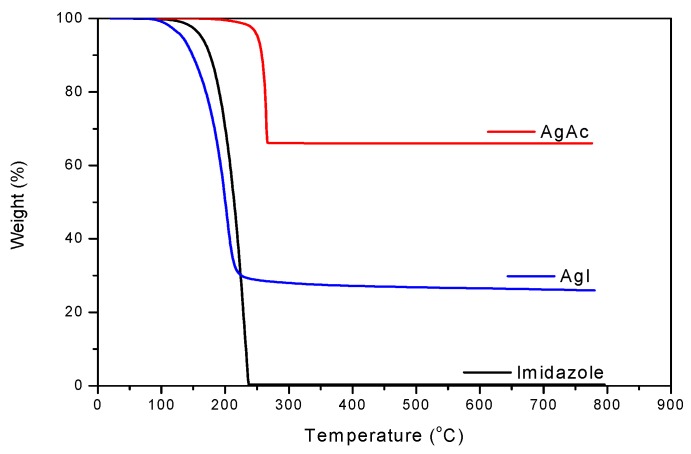
Thermogravimetric analysis thermograms of imidazole, silver–imidazole complex (AgI), and silver acetate (AgAc).

Figure 1 displays a TEM micrograph and a histogram for the diameter of the AgNWs synthesized via the microwave process. The average diameter of the NWs was 80 nm. Figure 2 shows the SEM image and a histogram for the length of AgNWs after purification for removing the AgNPs from the synthesized sample. The average length of the NWs was 12.5 µm. Table 1 shows the EDX data for the cured epoxy/silver nanocomposite with a 10 wt % silver–imidazole complex. The epoxy/silver nanocomposite cured with the silver–imidazole complex had 3.29 wt % silver particles. The silver–imidazole-cured epoxy nanocomposite contained silver atoms. In contrast, the imidazole-cured nanocomposite did not contain silver atoms. Figure 3 shows the TGA thermograms of the imidazole, AgAc, and the silver–imidazole complex. It should be noted that 26 wt % silver was formed by the decomposition of the silver–imidazole complex. It was assumed that the AgNPs were formed *in situ* via the decomposition of the silver–imidazole complex during the curing of the epoxy resin. It is of interest to note that the silver content measured by EDX for the epoxy resin cured with the 10 wt % silver–imidazole complex was 3.29 wt %, which was higher than that expected, *i.e.*, 2.6 wt %, considering that 26 wt % silver was formed by the decomposition of the silver–imidazole complex in TGA. It is speculated that the silver content measured by EDX reflects silver atoms in the surface of protruded silver nanoparticles in the fracture surface and can be higher than the silver content based on the TGA thermogram.

**Figure 4:**
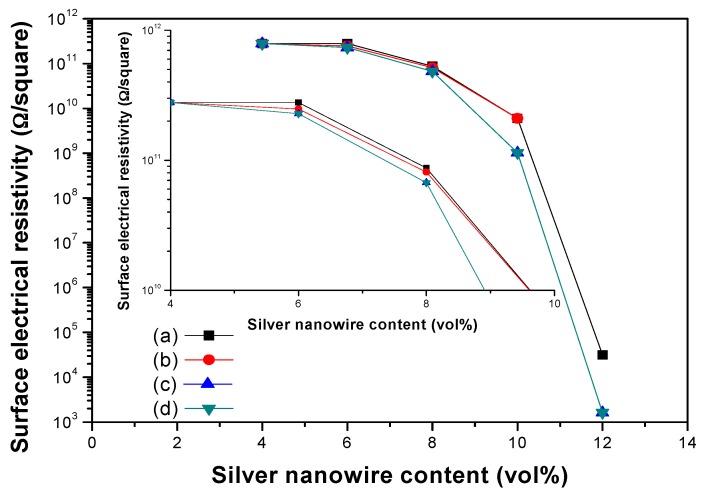
Surface electrical resistivity of the cured epoxy resin containing AgNWs of various concentrations: (**a**) sample cured with imidazole in the direction of the highest resistivity; (**b**) sample cured with imidazole in the direction perpendicular to that of (**a**); (**c**) sample cured with silver–imidazole complex in the direction of the highest resistivity; (**d**) sample cured with silver–imidazole complex in the direction perpendicular to that of (**c**).

Figure 4 shows the surface electrical resistivities of the nanocomposites containing AgNWs at various concentrations with the imidazole and silver–imidazole complexes. Generally, the silver–imidazole-cured samples had lower surface electrical resistivities when compared with those of the imidazole-cured samples. Corcione and Maffezzoli measured transport properties of graphite/epoxy composites and characterized them with various theoretical models [29]. As the electrical resistivity of the nanocomposites in Figure 4 showed threshold decrease with increasing AgNW contents, the statistical percolation model was employed in this study. Thus, surface electrical resistivity (σ) can be expressed as follows:

σ = *k*(*P* − *P*_c_)^−*t*^ (1)

where *P* is the volume concentration of AgNW, *P*_c_ the critical volume concentration for the percolation threshold of AgNW, and *t* the exponent related to the dimensionality of the system. Fittings to Equation (1) were carried out as shown in Figure S2 of Supplementary Materials. Parameters obtained by the fitting are given in Table 2. It was observed that *P*_c_ values of the epoxy/AgNW nanocomposite with *in situ*-formed silver nanoparticles was lower than that of the epoxy/AgNW nanocomposite without the *in situ*-formed silver nanoparticles. It may be noted that the *in situ*-formed AgNP exerted a beneficial effect on the epoxy/silver nanocomposites in terms of electrical properties. The *in situ*-formed AgNPs between AgNWs decreased the interparticle distance, which facilitated the transfer of electrons between the AgNWs and AgNPs. The imidazole-cured samples had higher surface electrical resistivity when compared with those of the silver–imidazole-cured samples with the same AgNW volume fraction. This was due to the absence of silver nanoparticles formed by the *in situ* process. The anisotropic property, resulting from the characteristic high aspect ratio of the AgNW, was confirmed by the surface electrical resistivity in the imidazole-cured samples. This phenomenon was especially observed in AgNWs at the loading of 6 to 10 vol %. However, it was not observed in AgNWs below 6 vol % or over 10 vol %. However, the silver–imidazole-cured nanocomposite did not exhibit the anisotropic phenomenon because of the presence of the silver nanoparticles formed by the *in situ* process. This is an important advantage of the nanocomposites composed of the *in situ*-formed silver nanoparticles as well as the AgNW. This allows the potential applications of these nanocomposites in devices requiring isotropic electrical properties.

**Figure 5:**
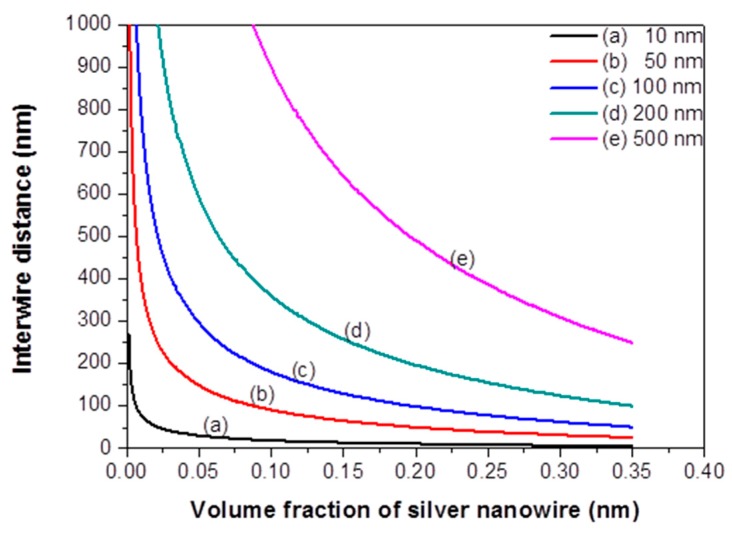
Interwire distances of AgNWs of different diameters: (**a**) 10 nm; (**b**) 50 nm; (**c**) 100 nm; (**d**) 200 nm; and (**e**) 500 nm.

Figure 5 shows the interwire distance in AgNW systems obtained from a geometrical model as described below. The interwire distance is defined as the distance between the two NW surfaces and is useful, as it can be used in conjunction with potential energy curves to determine whether attractive or repulsive energy dominates in a NW particulate system. This information is useful in inferring the stability of NW systems and other surface-related phenomena. Interwire distance has been used as an important parameter for the viscosity estimation of suspensions such as in metallic filler/epoxy resin systems [30]. It also provides useful insights into problems related to formulating suspensions with high filler loadings. At the maximum filler loading, the interwire distance tends to approach a minimum value. Thus, it is useful to understand how the interwire distance affects the physical characteristics of the NW particulates. The model assumes that a parallel piped cell surrounds each NW. It also assumes that the NW volume fraction in a cell is equal to the NW volume fraction throughout the system. Given that the NW is cylindrical, the NW diameter is *d*_w_, and the interwire distance of each NW is *d*_i_. The volume fraction of the NWs is Φ_w_, as given by:

Φ_w_ = 𝑉_w_/𝑉_u_ (2)

where 𝑉_w_ is volume of NWs in the nanocomposite volume of which is 𝑉_u_, and can be computed as:

𝑉_w_ = *n*π(𝑑_w_/2)^2^𝐿 (3)

𝑉_u_ = *n*(𝑑_i_ + 𝑑_w_)^2^𝐿 (4)

Equations (2)–(4) are combined to yield *d_i_* as a function of Φ_w_ as follows:

𝑑_i_ = 𝑑_w_[{π/(4Φ_w_)}^1/2^ − 1] (5)

The interwire distance for a AgNW of diameter 20 nm and volume fraction of 10 vol % was similar to that of the AgNW system with a NW diameter of 200 nm and a NW volume fraction of 20 vol %. This clearly indicated that an increase in the AgNW diameter increased the amount of AgNW dispersed into a medium and formed a high solid loading suspension. However, as the AgNW diameter decreased, the suspension was reduced to clumps. As the interwire distance is very small, it does not allow any more AgNWs to be dispersed into the system, even at a very low AgNW volume fraction. This may be the reason why it is extremely difficult to obtain a high solid loading suspension when the AgNW diameter size is in the nanometer range. The interwire distance in both the 10 vol % of the AgNW imidazole-cured sample and the silver–imidazole-cured sample in this study should be 144 nm.

**Figure 6:**
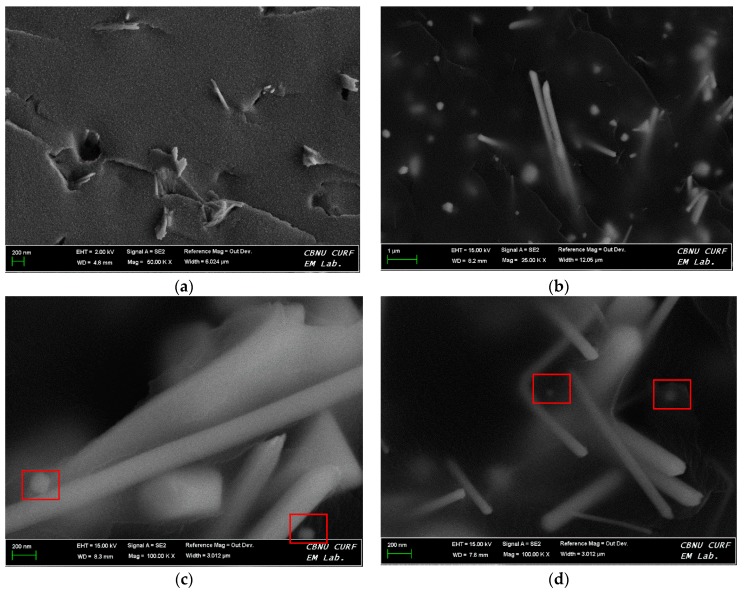
TEM images of the cured epoxy resin/silver nanocomposites: (**a**) sample cured with imidazole (scale bar, 200 nm); (**b**) sample cured with 10.0 wt % of the silver–imidazole complex with 6 vol % of AgNWs (scale bar, 1 μm); (**c**) magnified version of the sample in (**b**) (scale bar, 200 nm); (**d**) sample cured with 10.0 wt % of silver–imidazole with 8 vol % of AgNWs (scale bar, 200 nm).

The morphology of the cured systems was investigated using SEM. Figure 6a shows the SEM images of the films cured with imidazole with 8 vol % of AgNWs. No particles are observed in Figure 6a. Figure 6b,c show the SEM images of the films cured using 10.0 wt % of the silver–imidazole complex with 6 vol % of AgNWs. The *in situ*-formed nanoparticles were well dispersed in the epoxy resin with AgNWs, and no macroscopic agglomerates were formed. The average particle diameter was ~100 nm. The quality of the AgNW dispersion in the polymer matrix was directly correlated with its effectiveness in improving the properties of the nanocomposites. It is believed that a uniform distribution of the *in situ*-formed AgNPs in the polymer matrix with AgNWs leads to a relatively high electrical conductivity. The addition of AgNWs in conjunction with the *in situ* formation of AgNPs facilitated a more uniform dispersion of the NPs in the polymers, thereby reducing anisotropic properties.

## 4. Conclusions

Epoxy/silver nanocomposites were successfully prepared by the dispersion of AgNWs and the *in situ* formation of AgNPs via the thermal decomposition of a silver–imidazole complex. The simultaneously released imidazole acted as a catalyst to help cure the epoxy resin. The molar ratio of AgAc to imidazole in the silver–imidazole complex was 1:2. AgNPs were formed *in situ* during the curing process. The average particle diameter was ~100 nm. The *in situ*-formed AgNPs in the nanocomposites altered the electrical properties of the nanocomposites from anisotropic to isotropic. The nanocomposites of epoxy resin and the AgNWs with isotropic electrical properties caused by the *in situ* formation of AgNPs are useful as electronic materials in various applications.

## Figures and Tables

**Table 1:** Energy-dispersive X-ray spectroscopy data for epoxy/silver nanocomposites cured with the 10 wt % silver–imidazole complex and the epoxy resin cured with imidazole.

Element	Epoxy Resin Cured with the Silver–Imidazole Complex		Epoxy Resin Cured with Imidazole	
	Wt %	At %	Wt %	At %
C	67.86	75.50	69.48	75.62
O	28.85	24.09	30.52	24.38
Ag	3.29	00.41	0.00	0.00

**Table 2:** Parameter values of the percolation model for samples shown in Figure 4.

Sample	(a)	(b)	(c)	(d)
***P*_c_** **(vol %)**	12.60	12.80	12.50	12.50
***t***	4.29	4.57	4.49	4.49

## Supplementary Materials

The following are available online at www.mdpi.com/2073-4360/8/4/157/s1. Figure S1: DSC thermograms obtained by scanning at heating rate of 10 °C/min in N_2_ gas environment for the epoxy resins containing anhydride type curing agent: (**a**) without silver-imidazole complex; (**b**) with 10 wt % of the silver-imidazole complex. Exothermic heat of cure in (**b**) was 321.3 J/g, Figure S2: Fittings to the percolation model Equation (1) for surface electrical resistivity of the samples shown in Figure 4: (**a**) sample cured with imidazole in direction of the highest resistivity; (**b**) sample cured with imidazole in the direction perpendicular to that of (**a**); (**c**) sample cured with silver-imidazole complex in direction of the highest resistivity; (**d**) sample cured with silver-imidazole complex in the direction perpendicular to that of (**c**).
