# Supplementary material for: The Effects of in Situ-Formed Silver Nanoparticles on the Electrical Properties of Epoxy Resin Filled with Silver Nanowires
Source: Polymers (Basel). 2016 Apr 21;8(4):157. doi: 10.3390/polym8040157 (PMC6432508; doi:10.3390/polym8040157)
Supplement: Supplementary file 1 [file polymers-08-00157-s001.pdf]

Polymers **2016**, *8*, 157; doi:10.3390/polym8040157

Supplementary Material: Effects of *in Situ* Formed Silver Nanoparticles on Electrical Properties of Epoxy Resin Filled with Silver Nanowire

Gwang-Seok Song, Dai-Soo Lee and Ilho Kang

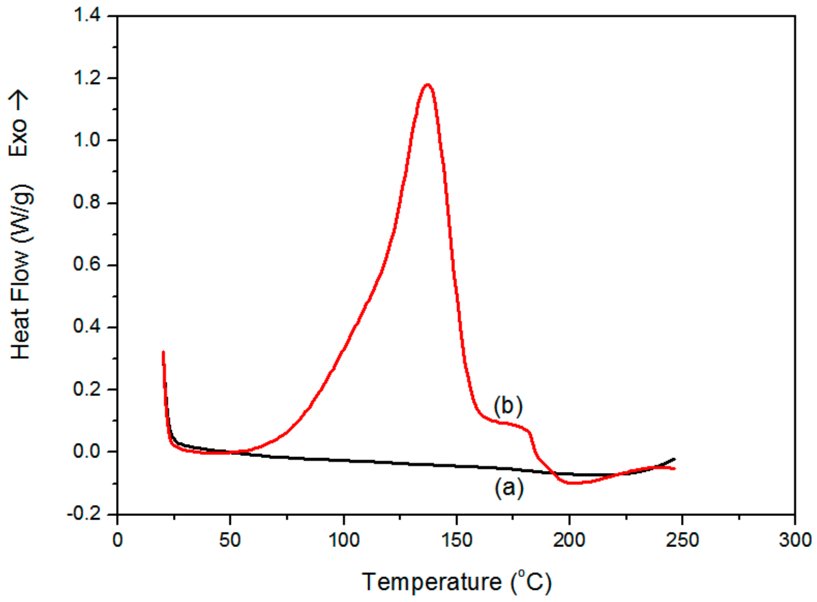

Figure S1. DSC thermograms obtained by scanning at heating rate of 10 °C/min in N_2 gas environment for the epoxy resins containing anhydride type curing agent: (**a**) without silver-imidazole complex; (**b**) with 10 wt % of the silver-imidazole complex. Exothermic heat of cure in (**b**) was 321.3 J/g.

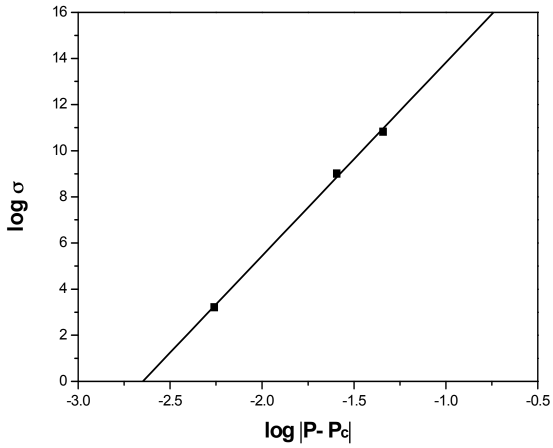

(**a**)

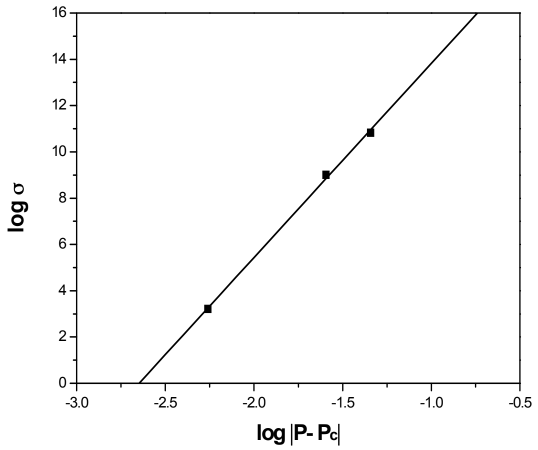

(**b**)

Figure S2. *Cont.*

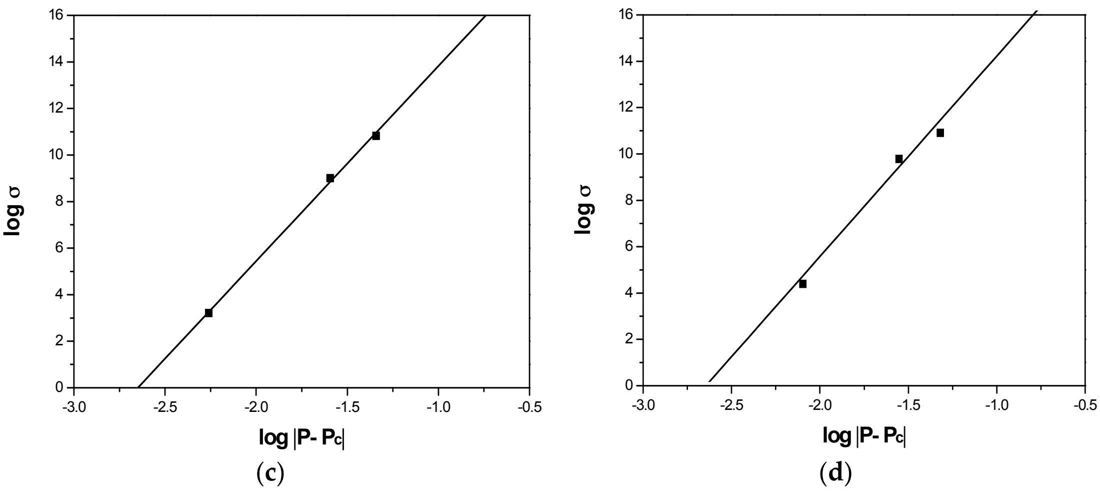

Figure S2. Fittings to the percolation model Equation (1) for surface electrical resistivity of the samples shown in Figure 4: (**a**) sample cured with imidazole in direction of the highest resistivity; (**b**) sample cured with imidazole in the direction perpendicular to that of (**a**); (**c**) sample cured with silver-imidazole complex in direction of the highest resistivity; (**d**) sample cured with silver-imidazole complex in the direction perpendicular to that of (**c**).

© 2016 by the authors; licensee MDPI, Basel, Switzerland. This article is an open access article distributed under the terms and conditions of the Creative Commons by Attribution (CC-BY) license (http://creativecommons.org/licenses/by/4.0/).
